# miR-378a-3p inhibits ischemia/reperfusion-induced apoptosis in H9C2 cardiomyocytes by targeting TRIM55 via the DUSP1-JNK1/2 signaling pathway

**DOI:** 10.18632/aging.103106

**Published:** 2020-05-28

**Authors:** Jiaying Tan, Jun Shen, Huigeng Zhu, Ye Gong, Hechen Zhu, Junping Li, Shan Lin, Gang Wu, Tao Sun

**Affiliations:** 1 Department of Critical Care Medicine, Huashan Hospital, Fudan University, Shanghai 200040, P.R. China; 2 Department of Gynaecology and Obstetrics, Huashan Hospital North, Fudan University, Shanghai 200040, P.R. China; 3 Department of Cardiology, Huashan Hospital, Fudan University, Shanghai 200040, P.R. China

**Keywords:** ischemia/reperfusion, miR-378a-3p, TRIM55, DUSP1, apoptosis

## Abstract

MicroRNAs (miRNAs) are involved in many pathological and biological processes, such as ischemia/reperfusion (I/R) injury by modulating gene expression. Increasing evidence indicates that miR-378a-3p might provide a potential cardioprotective effect against ischemic heart disease. Cell apoptosis is a crucial mechanism in I/R injury. As such, this study evaluated the protective effects and underlying mechanisms of action of miR-378a-3p on H9C2 cardiomyocyte apoptosis following I/R injury. We found that I/R-induced H9C2 cardiomyocytes exhibited a decrease in miR-378a-3p expression, while treatment with a miR-378a-3p mimic suppressed cell apoptosis, JNK1/2 activation, cleavage of PARP and caspase-3, and Bax/Bcl-2 ratio but increased DUSP1 expression, which subsequently inhibited JNK1/2 phosphorylation. TRIM55 was shown to be a target of miR-378a-3p and its downregulation inhibited the miR-378a-3p inhibitor-induced increase in cell apoptosis and JNK1/2 activation. TRIM55 inhibited DUSP1 protein expression through ubiquitination of DUSP1. Moreover, DUSP1 overexpression inhibited the TRIM55 overexpression-induced increase in cell apoptosis and JNK1/2 activation. The protective effect of miR-378a-3p was subsequently confirmed in a rat myocardial I/R model, as evidenced by a decrease in cardiomyocyte apoptosis of cardiomyocytes, TRIM55 expression, and JNK1/2 activation. Taken together, these results suggest that miR-378a-3p may protect against I/R-induced cardiomyocyte apoptosis via TRIM55/DUSP1/JNK signaling.

## INTRODUCTION

Ischemic heart disease is the leading cause of death in humans worldwide and the morbidity and mortality caused by myocardial ischemia have increased yearly. Currently, clinical treatments such as thrombolytic therapy or early coronary intervention in the ischemic site can effectively improve myocardial ischemia or necrosis [1, 2]. However, a long-term ischemia-reperfusion (I/R) could result in malignant arrhythmia, myocardial apoptosis, and acute hemodynamic disorder, which may lead to the occurrence and development of myocardial I/R injury [3, 4]. The mechanism of I/R injury involves various processes, including apoptosis, autophagy, oxidative stress, and inflammation [5, 6], among which cardiomyocyte apoptosis is the main characteristic of the disease and determines the severity of myocardial I/R injury. Therefore, inhibiting I/R-induced cardiomyocyte apoptosis may serve as a therapeutic strategy in myocardial I/R injury.

MicroRNAs (miRNA, miR) are highly conserved endogenous small non-coding RNAs which are involved in the post-transcriptional regulation of genes and play important roles in cell proliferation, differentiation, and apoptosis, as well as oxidative stress [7]. Increasing evidence suggests that miRNAs are associated with the occurrence and development of cardiovascular diseases by targeting different genes. For example, miR-497 was shown to suppress apoptosis and promote the proliferation of I/R-induced cardiomyocytes by targeting Mfn2 [8], while miR-208a enhanced the apoptosis of I/R-induced cardiomyocytes by targeting CHD9 [9]. Additionally, miR-378 was found to be downregulated during the hypertrophic growth of the heart and heart failure [10], while its upregulation attenuated cardiac hypertrophy and improved cardiac function by targeting MAPK1, IGF1R, GRB2, and KSR1 [11]. SAMD1, FSTL1, MAPK1 and NPAS4, which are putative downstream target genes of miR-378a-3p that have been confirmed to participate in cardiomyocyte apoptosis and I/R injury [12–15]. However, the role of miR-378a-3p in the regulation of apoptosis during myocardial I/R injury remains unclear.

The tripartite motif (TRIM) protein family, which are a family of ubiquitin ligases (E3), has been shown to be involved in cardiac pathophysiology, including dilated cardiomyopathy, cardiac ischemia/atrophy/hypertrophy, and cardiomyocyte apoptosis and differentiation [16]. Moreover, studies have shown that TRIM77 may protect against ischemic heart disease-associated myocardial infarction and the recombinant human TRIM77 protein was also shown to have cardioprotective effects against I/R injury [17]. TRIM54-/- mice were also found to be more prone to cardiac rupture after acute myocardial infarction [18]. However, although TRIM55 is sufficient for normal cardiac function and simultaneous absence of TRIM55 and TRIM63 results in physiological cardiac hypertrophy [19], its role in myocardial I/R injury is still not elucidated.

c-Jun N-terminal kinase (JNK), also known as stress-activated protein kinase (SAPK), is a member of the mitogen-activated protein kinase (MAPK) signaling pathway, which is associated with oxidative stress, endoplasmic reticulum stress, mitochondrial dysfunction, and apoptosis [20]. JNK activation has been found in H9C2 cardiomyocytes following hypoxia/reoxygenation injury [21] and was shown to increase the cleavage of PARP and caspase-3 [22], trigger apoptosis, and aggravate myocardial I/R injury by increasing the Bax/Bcl-2 ratio [23]. Additionally, studies have shown that dual-specificity protein phosphatase 1 (DUSP1) may dephosphorylate and inactivate JNK, and therefore alleviate cell apoptosis in cardiac I/R injury via the JNK pathway [24], resulting in a survival advantage to the myocardial tissue following I/R. However, the regulation of DUSP1/JNK signaling-associated apoptosis pathway in myocardial I/R injury is still unknown.

Our study aimed to investigate the effect of miR-378a-3p and TRIM55 on the apoptosis of cardiomyocytes following I/R injury *in vitro* and *in vivo* and the molecular mechanisms involved in the DUSP1/JNK signaling-associated apoptosis pathway. The observations in our study suggest that miR-378a-3p has a cardioprotective effect against myocardial I/R injury.

## RESULTS

### miR-378a-3p is downregulated in I/R-induced H9C2 cardiomyocytes and inhibits cell apoptosis

**Figure 1:**
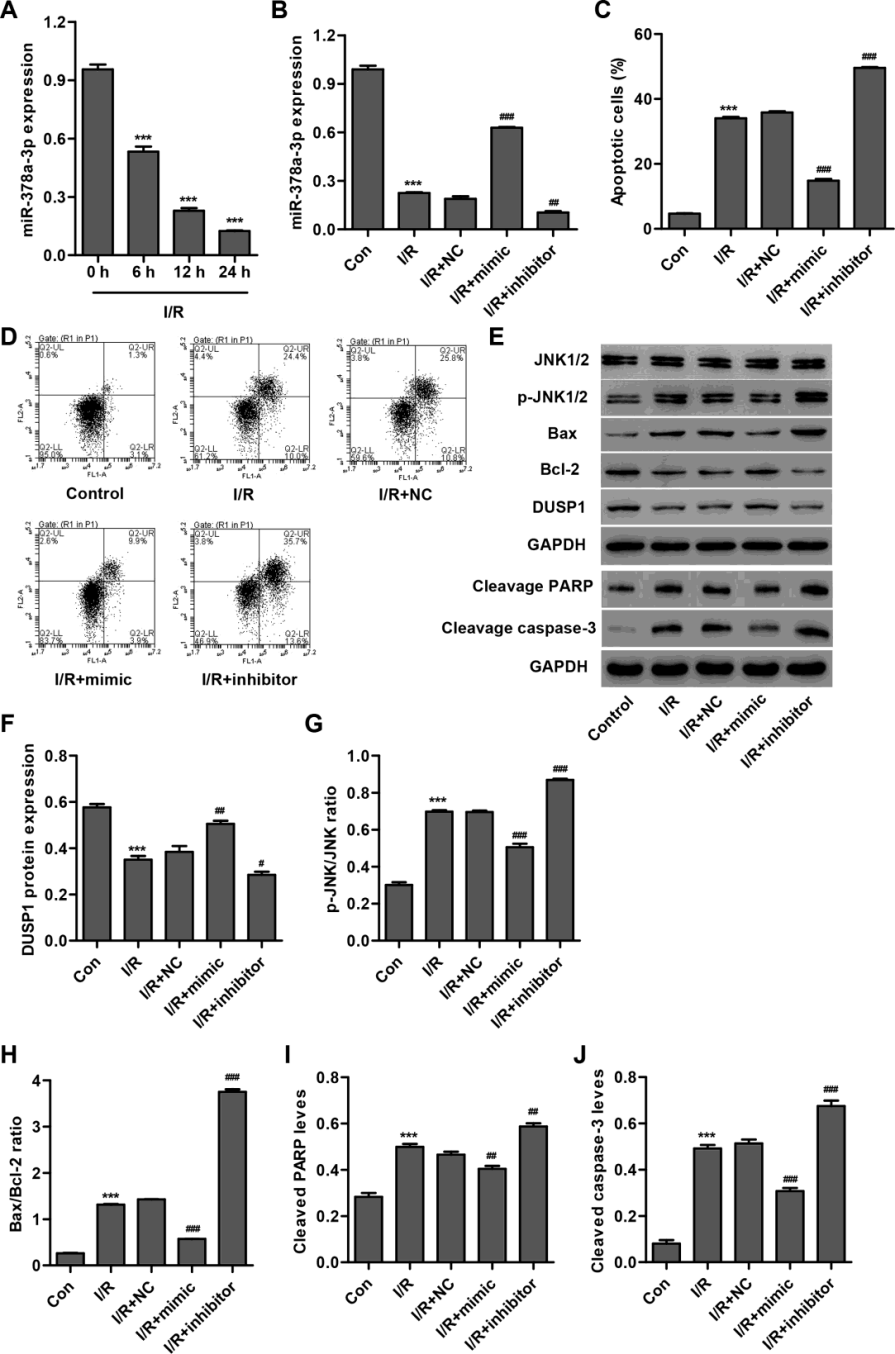
**miR-378a-3p is upregulated in I/R-induced H9C2 cardiomyocytes and inhibits cell apoptosis.** (**A**) miR-378a-3p expression was measured by Real-time PCR in H9C2 cardiomyocytes following 3 h ischemia and 6, 12 or 24 h reperfusion. H9C2 cardiomyocytes following 3 h ischemia and 24 h reperfusion were transfected with a miR-378a-3p mimic, inhibitor or negative control (NC) and those without I/R injury were used as a control. (**B**) miR-378a-3p expression, (**C**, **D**) cell apoptosis, and (**E**–**J**) protein expression of DUSP1, p-JNK1/2, JNK1/2, cleaved PARP and caspase-3, Bax, and Bcl-2 were measured. ***P < 0.001 compared with 0 h or I/R. ^#^*P* < 0.05, ^##^*P* < 0.01, ^###^*P*<0.001 compared with I/R + NC.

In order to examine the role of miR-378a-3p in cardiac I/R injury *in vitro*, we first measured miR-378a-3p expression in H9C2 cardiomyocytes following 3 h ischemia and 6, 12 or 24 h reperfusion (I/R injury). As shown in Figure 1A, miR-378a-3p expression in H9C2 cardiomyocytes following I/R injury was significantly decreased by 44.3%, 76.1%, and 86.9%, respectively, compared to the control, suggesting that miR-378a-3p may play a role in the development of I/R injury. Subsequently, a miR-378a-3p mimic and inhibitor were used to examine the function of miR-378a-3p in the apoptosis of H9C2 cardiomyocytes after 3 h ischemia and 24 h reperfusion (I/R). We found that I/R-induced H9C2 cardiomyocytes transfected with the miR-378a-3p mimic showed a significant increase in miR-378a-3p expression (2.31-fold), while transfection with the miR-378a-3p inhibitor led to a 44.7% decrease in miR-378a-3p expression when compared with the miR-378a-3p NC (Figure 1B). Moreover, H9C2 cardiomyocytes after I/R injury exhibited a 7.29-fold increase in cell apoptosis when compared with H9C2 cardiomyocytes without I/R injury (control) (Figure 1C, 1D). However, I/R-induced H9C2 cardiomyocytes transfected with a miR-378a-3p mimic showed a 58.6% decrease in cell apoptosis, while transfection with a miR-378a-3p inhibitor led to a 38.3% increase in cell apoptosis when compared to the NC.

To explore the underlying mechanism by which miR-378a-3p inhibits I/R-induced apoptosis of H9C2 cardiomyocytes, we measured the expression of DUSP1, p-JNK1/2, JNK1/2, cleaved PARP, cleaved, caspase-3, Bax, and Bcl-2 in I/R-induced H9C2 cardiomyocytes transfected with a miR-378a-3p mimic or inhibitor. Our results demonstrated that I/R injury significantly increased JNK1/2 activation, cleaved PARP and caspase-3 expression, and the Bax/Bcl-2 ratio and inhibited DUSP1 expression. Moreover, these effects were found to be inhibited by the miR-378a-3p mimic and strengthened by the miR-378a-3p inhibitor (Figure 1E–1J). Taken together, our results suggest that miR-378a-3p may inhibit I/R-induced apoptosis of H9C2 cardiomyocytes via DUSP1/JNK1/2 signaling.

### TRIM55 is a target of miR-378a-3p and its expression is upregulated in H9C2 cardiomyocytes following I/R injury

**Figure 2:**
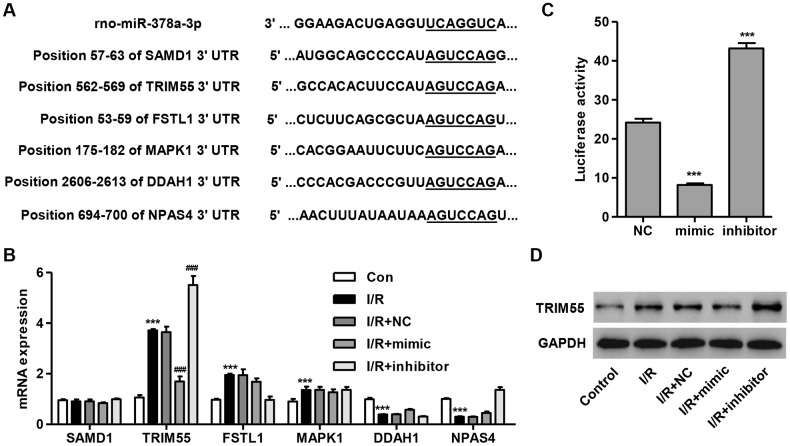
**TRIM55 is a target of miR-378a-3p.** (**A**) Complementary miR-378a-3p binding sequences in the 3′ UTR of SAMD1, TRIM55, FSTL1, MAPK1, DDAH1, and NPAS4. (**B**) SAMD1, TRIM55, FSTL1, MAPK1, DDAH1, and NPAS4 mRNA expression in H9C2 cardiomyocytes following I/R injury transfected with a miR-378a-3p mimic, inhibitor, or negative control (NC) was measured. (**C**) The dual-luciferase reporter assay was performed to confirm that TRIM55 is a target of miR-378a-3p. (**D**) H9C2 cardiomyocytes following 3 h ischemia and 24 h reperfusion were transfected with a miR-378a-3p mimic, inhibitor or NC and those without I/R injury were used as a control. Then, the protein expression of TRIM55 was measured. ***P < 0.001 compared with the control or NC. ^###^*P* < 0.001 compared with I/R + NC.

To determine the target gene of miR-378a-3p, we used a computational approach by utilizing TargetScan (http://www.targetscan.org/) to predict the downstream target genes of miR-378a-3p. The data showed that miR-378a-3p may bind to the 3′ UTR region of SAMD1, FSTL1, MAPK1, DDAH1, and NPAS4 (Figure 2A), which have been associated with I/R injury and apoptosis [12–15, 25]. Notably, TRIM72 has been shown to protect the myocardium following I/R injury [17]. Therefore, TRIM55 was also investigated as a putative target of miR-378a-3p. We demonstrated that TRIM55 mRNA expression was upregulated in I/R-induced H9C2 cardiomyocytes and was further upregulated by the miR-378a-3p inhibitor and suppressed by the miR-378a-3p mimic (Figure 2B). Next, a dual-luciferase reporter assay was performed to confirm that the TRIM55 mRNA is a target of miR-378a-3p (Figure 2C). Additionally, the TRIM55 protein expression was shown to be upregulated in H9C2 cardiomyocytes following I/R injury, an effect which was inhibited by treatment with the miR-378a-3p mimic and promoted by the miR-378a-3p inhibitor (Figure 2D). These results indicate that TRIM55 is a target of miR-378a-3p and may be involved in I/R-induced injury in H9C2 cardiomyocytes.

### TRIM55 silencing suppresses miR-378a-3p inhibitor-induced JNK1/2 activation and cell apoptosis

**Figure 3:**
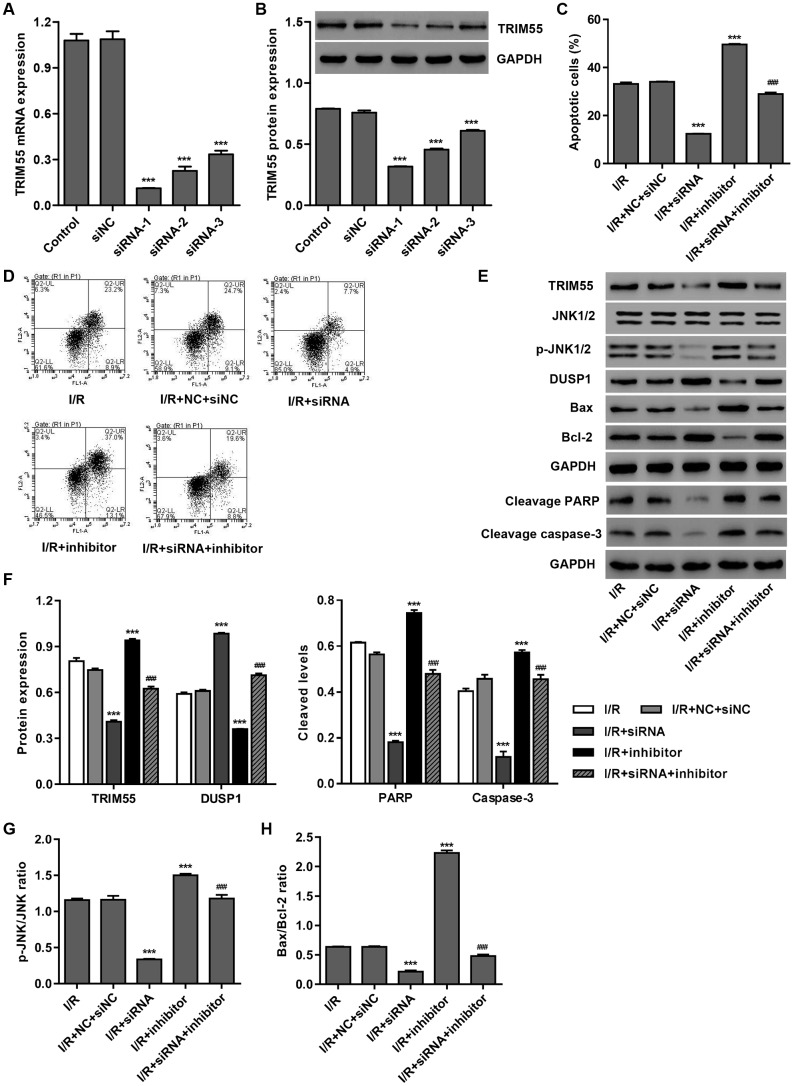
**TRIM55 silencing inhibits I/R- and miR-378a-3p inhibitor-induced apoptosis of H9C2 cardiomyocytes.** H9C2 cardiomyocytes were transfected with three TRIM55-siRNAs (siRNA-1, siRNA-2, siRNA-3) or scramble siRNA (siNC). (**A**, **B**) TRIM55 expression was measured. H9C2 cardiomyocytes following I/R injury were transfected with the TRIM55-siRNA and/or miR-378a-3p inhibitor. (**C**, **D**) Cell apoptosis was measured by flow cytometry. (**E**–**H**) Expression of TRIM55, DUSP1, JNK1/2, cleaved PARP and caspase-3, Bax, and Bcl-2 was measured. ***P < 0.001 compared with siNC or I/R + NC + siRNA. ^###^*P* < 0.001 compared with I/R + inhibitor.

To investigate the role of TRIM55 in miR-378a-3p-mediated apoptosis, TRIM55 was silenced in H9C2 cardiomyocytes following I/R injury. As shown in Figure 3A, 3B, siRNA-1, siRNA-2, and siRNA-3 significantly decreased TRIM55 mRNA levels by 89.7%, 79.2%, and 69.3%, and TRIM55 protein levels by 58.2%, 39.9%, and 19.7%, respectively, when compared to the siNC. Moreover, TRIM55 silencing significantly inhibited the cell apoptosis induced by I/R injury and transfection with the miR-378a-3p inhibitor (Figure 3C, 3D). Additionally, TRIM55 silencing significantly reduced TRIM55 expression, the cleavage of PARP and caspase-3, JNK1/2 activation, and Bax/Bcl-2 ratio induced by I/R injury and the miR-378a-3p inhibitor (Figure 3E–3H).

### TRIM55 overexpression promotes I/R-induced JNK1/2 activation and cell apoptosis via ubiquitination of DUSP1

**Figure 4:**
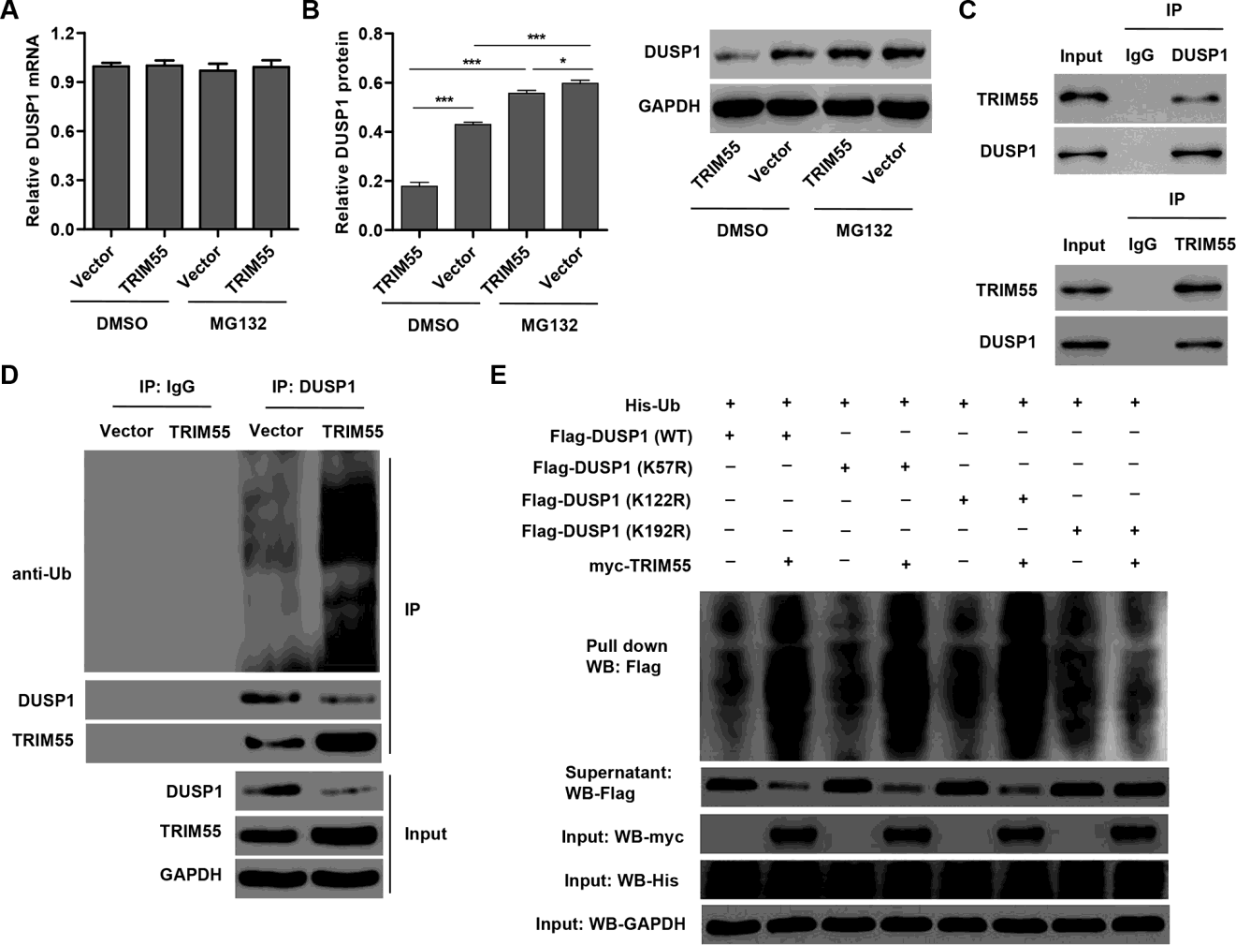
**TRIM55 interacts with and induces ubiquitination of DUSP1.** (**A**, **B**) H9C2 cardiomyocytes were transduced with a TRIM55 expression vector or blank vector in the absence or presence of MG132 and then the expression of DUSP1 was measured. (**C**) H9C2 cardiomyocytes lysates were subjected to immunoprecipitation with control IgG, anti-DUSP1 or anti-TRIM55 antibody. The immunoprecipitates were then blotted with the indicated antibodies. (**D**) H9C2 cardiomyocytes transduced with a TRIM55 expression vector or blank vector were immunoprecipitated with anti-DUSP1, followed by immunoblotting with indicated antibodies. (**E**) H9C2 cardiomyocytes were co-transfected with a DUSP1 (WT) or mutant DUSP1 constructs along with the myc-TRIM55 and His-Ubiquitin constructs and then a pull-down assay was carried out. *P < 0.05, ***P < 0.001.

In view of the role of miR-378a-3p in regulating DUSP1 expression and JNK1/2 activation in I/R-induced H9C2 cardiomyocytes, we hypothesized that TRIM55, as an E3 ubiquitin ligase, may participate in this process. Our data showed that TRIM55 overexpression had no effect on the mRNA expression of DUSP1 (Figure 4A) but decreased DUSP1 protein expression (Figure 4B), which was reversed by treatment with the proteasome inhibitor MG132. This suggests that TRIM55 may be involved in the post-transcriptional regulation of DUSP1. Co-immunoprecipitation and ubiquitination analysis showed that TRIM55 interacted with DUSP1 and induced DUSP1 ubiquitination (Figure 4C, 4D). Moreover, the results of the pull-down assay indicated that K192 is required for TRIM55-induced ubiquitination of DUSP1 (Figure 4E).

**Figure 5:**
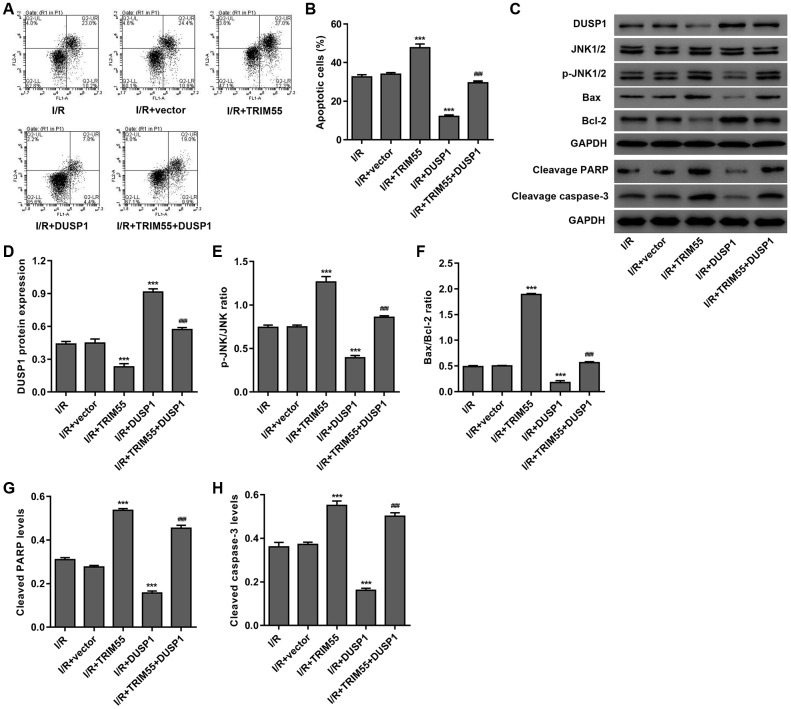
**DUSP1 overexpression inhibits I/R- and TRIM55 overexpression-induced apoptosis of H9C2 cardiomyocytes.** H9C2 cardiomyocytes following I/R injury were transduced with a TRIM55 and/or DUSP1 expression vector or blank vector. (**A**, **B**) Cell apoptosis was measured by flow cytometry. (**C**–**H**) The expression of DUSP1, JNK1/2, JNK1/2, cleavage of PARP and caspase-3, Bax, and Bcl-2 was also measured. ***P < 0.001 compared with I/R + vector. ^###^*P* < 0.001 compared with I/R + TRIM55.

To further investigate the role of DUSP1 in TRIM55-induced I/R injury, a TRIM55 and/or DUSP1 expressing vector was transduced into H9C2 cardiomyocytes following I/R injury. As shown in Figure 5A–5H, DUSP1 overexpression significantly reduced cell apoptosis, cleavage of PARP and caspase-3, JNK1/2 activation, and Bax/Bcl-2 ratio induced by I/R injury and TRIM55 overexpression. Taken together, our results indicate that TRIM55 may promote I/R-induced apoptosis of H9C2 cardiomyocytes via ubiquitination of DUSP1.

### miR-378a-3p inhibits apoptosis in I/R-induced rats

**Figure 6:**
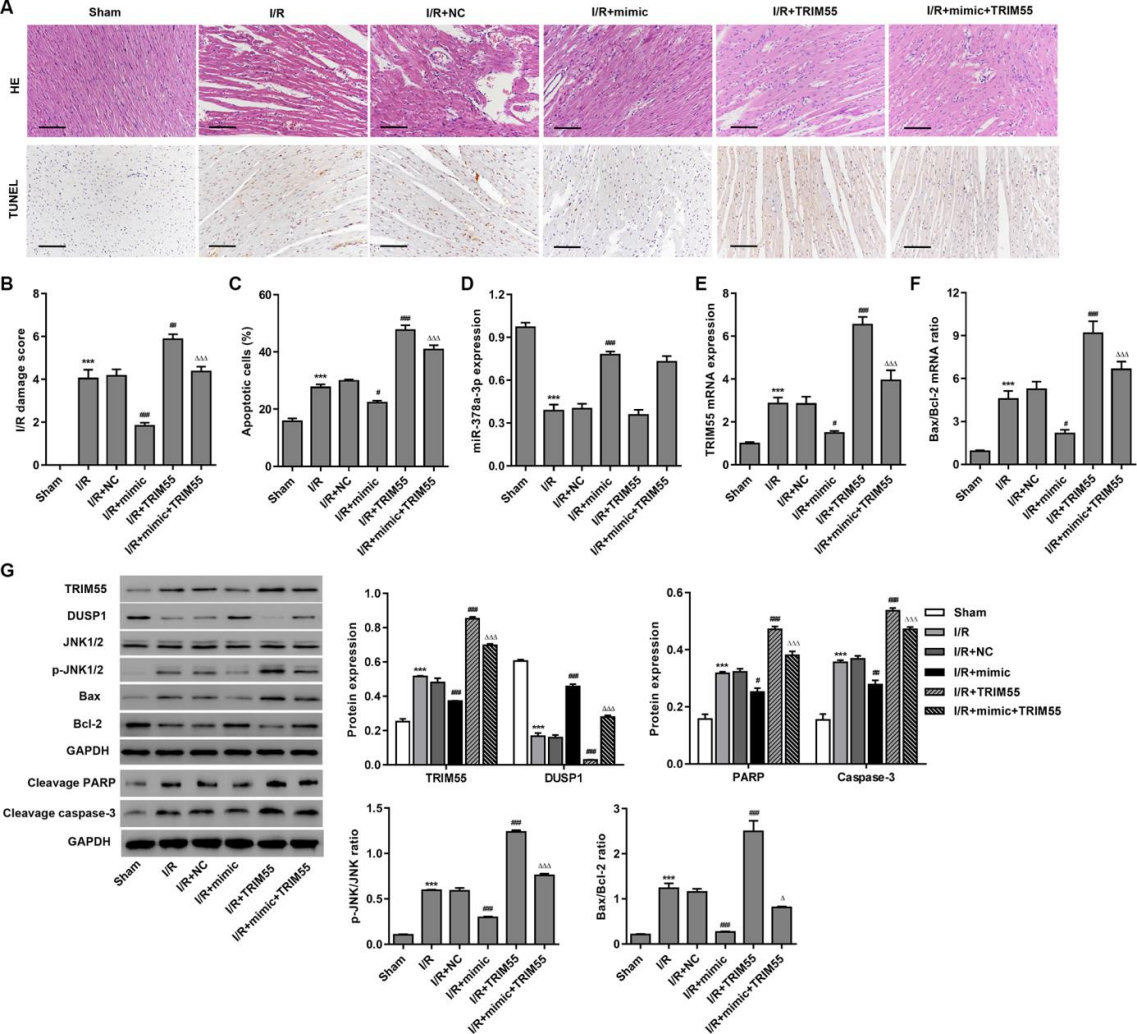
**The miR-378a-3p mimic inhibits I/R-induced apoptosis in rats.** Myocardial I/R model rats were injected with 50 mg/kg of the miR-378a-3p mimic or negative control (NC) 24 h before LCA ligation. (**A**–**C**) Histological assessment of the myocardium was performed by H&E staining and TUNEL. Scale bar: 100 μm. (**D**–**F**) The expression of miR-378a-3p, TRIM55, Bax, and Bcl-2 was measured by real-time PCR. (**G**) The expression of TRIM55, DUSP1, p-JNK1/2, JNK1/2, cleavage of PARP and caspase-3, Bax, and Bcl-2 was measured by western blotting. ***P < 0.001 compared with sham. ^#^*P* < 0.05, ^##^*P* < 0.01, ^###^*P* < 0.001 compared with I/R + NC. ^Δ^*P* < 0.05, ^ΔΔΔ^*P* < 0.001 compared with I/R + mimic.

To further examine the role of miR-378a-3p in I/R-induced cardiomyocyte apoptosis *in vivo*, myocardial I/R rats were injected with a miR-378a-3p mimic or NC 24 h prior to LCA ligation. The histological assessment showed that treatment with the miR-378a-3p mimic markedly suppressed I/R-induced injury and cardiomyocyte apoptosis (Figure 6A–6C). Furthermore, pre-treatment with the miR-378a-3p mimic markedly increased miR-378a-3p and DUSP1 expression and decreased TRIM55 expression, JNK1/2 activation, cleavage of PARP and caspase-3, and Bax/Bcl-2 ratio in the myocardial tissue following I/R injury (Figure 6D–6G). However, TRIM55 overexpression was found to promote I/R-induced JNK1/2 activation and cell apoptosis but inhibit the miR-378a-3p mimic-induced decrease in JNK1/2 activation and cell apoptosis. As such, these data further support our findings in H9C2 cardiomyocytes with I/R injury.

## DISCUSSION

In the present study, we showed that miR-378a-3p is downregulated in H9C2 cardiomyocytes after a simulated I/R injury and that its upregulation suppressed JNK1/2 activation and cell apoptosis induced by I/R injury. Furthermore, we provided evidence for the critical role of TRIM55 as a target of miR-378a-3p and shoed that it was associated with JNK1/2 activation and cell apoptosis induced by I/R injury and treatment with a miR-378a-3p inhibitor via ubiquitination of DUSP1.

MiRNAs play an important role in the regulation of cardiomyocyte differentiation and apoptosis. In myocardial I/R injury, abnormal miRNA expression is considered to be a diagnostic biomarker and also a potential therapeutic target. Moreover, in a myocardial infarction model, miR-150, miR-34a, and miR-130a were found to be closely related to the occurrence of myocardial I/R injury [26]. MiR-497 was shown to be downregulated in myocardial tissues after myocardial I/R injury and inhibit cardiomyocyte apoptosis [8], whereas miR-208 was found to be upregulated in H9C2 cardiomyocytes after simulated I/R injury and promote cell apoptosis [9]. These results suggest that myocardial I/R injury is closely related to abnormal miRNA expression. Herein, miR-378a-3p was found to be downregulated in I/R-induced H9C2 cardiomyocytes in a time-dependent manner, while treatment with a miR-378a-3p mimic suppressed cell apoptosis both *in vitro* and *in vivo*. Moreover, treatment with a miR-378a-3p inhibitor showed an inverse effect in H9C2 cardiomyocytes. Our findings suggest that miR-378a-3p may be involved in the apoptosis of cardiomyocytes following cardiac I/R injury. Similar to our findings, other studies have shown that miR-378a-3p upregulation also attenuated cardiac hypertrophy and improved cardiac function [10, 11].

SAMD1, FSTL1, MAPK1, DDAH1, and NPAS4, which are putative downstream target genes of miR-378a-3p, were previously shown to play a role in I/R injury and cardiomyocyte apoptosis [12–15, 25]. Moreover, the TRIM family of proteins, including TRIM72 and TRIM54, were also shown to be associated with cardiac I/R injury. TRIM55, which is a putative downstream target gene of miR-378a-3p, was also associated with normal cardiac function and physiological cardiac hypertrophy [19]. We confirmed that only the expression of the TRIM55 mRNA was decreased by treatment with the miR-378a-3p mimic and increased by the miR-378a-3p inhibitor. Moreover, we showed that its expression was upregulated in I/R-induced H9C2 cardiomyocytes. TRIM55 silencing also inhibited cell apoptosis induced by I/R injury and treatment with the miR-378a-3p inhibitor. As such, our data suggest that miR-378a-3p may inhibit cardiac I/R injury by targeting TRIM55.

The TRIM protein family is a family of E3 ubiquitin ligases, including TRIM31 [27], TRIM52 [28], TRIM50 [29], and TRIM18 [30], and were found to be related to a broad range of biological and pathological processes via ubiquitination. TRIM55 was also identified as the first ubiquitin ligase to inhibit PPAR expression via post-translational ubiquitination and to protect against diabetic cardiomyopathy [31]. Our data also demonstrated that TRIM55 overexpression induced ubiquitination of DUSP1, whereas JNK1/2 activation and cell apoptosis induced by TRIM55 overexpression was inhibited by DUSP1 overexpression. This suggests that TRIM55 may play a role in mediating cardiac I/R injury via DUSP1 regulation. Studies have indicated that DUSP1 may alleviate cardiac I/R injury by inhibiting JNK activation [24], suggesting its important role in myocardial I/R injury. Herein, TRIM55 silencing inhibited cell apoptosis and JNK1/2 activation induced by I/R injury and treatment with the miR-378a-3p inhibitor. SP600125, a JNK inhibitor, was found to inhibit JNK activation and cleavage of PARP and caspase-3 [22], as well as the cardiac I/R-induced increase in the Bax/Bcl-2 ratio both *in vitro* and *in vivo* [32, 33]. Therefore, we hypothesize that TRIM55 may promote cell apoptosis in cardiac I/R injury via ubiquitination of DUSP1, followed by activation of JNK1/2 signaling, which then leads to an increase in PARP and caspase-3 cleavage, as well as the Bax/Bcl-2 ratio.

In conclusion, our data suggest that downregulation of miR-378a-3p contributes to cardiac I/R injury. MiR-378a-3p upregulation exerts a protective effect against I/R-induced H9C2 cardiomyocyte apoptosis and JNK1/2 activation by targeting TRIM55. TRIM55 interacts with and induces ubiquitination of DUSP1, which may be involved in the miR-378a-3p-mediated JNK1/2 activation and cell apoptosis in cardiac I/R injury. Thus, TRIM55 may represent a potential therapeutic target for myocardial I/R injury.

## MATERIALS AND METHODS

### Reagents and antibodies

Dulbecco’s Modified Eagle’s Medium (DMEM) and fetal bovine serum (FBS) were purchased from Gibco Lab. (Grand Island, NY, USA). The penicillin-streptomycin solution (100 ×) and RIPA buffer were from Solarbio (Beijing, China). The ECL kit was from Pierce Chemical Co (Rockford, IL, USA). Lipofectamine 2000, TRIzol reagent, Maxima SYBR Green/ROX qPCR Master Mix, and BCA Protein Assay kit were from Thermo Fisher Scientific (Waltham, MA, USA). The M-MLV Reverse Transcriptase was from Promega (Fitchburg, WI, USA). The anti-TRIM55 antibody was from Sigma (Shanghai, China). The anti-DUSP1, cleaved caspase-3, cleaved PARP, and anti-ubiquitin antibodies were from Abcam (Cambridge, MA, USA). The anti-Bcl-2 and anti-Bax antibodies were from Santa Cruz Biotechnology (Dallas, TX, USA). The anti-p-JNK1/2, anti-JNK1/2, and anti-GAPDH antibodies were from Cell Signaling Technology (Danvers, MA, USA). The annexin V-FITC Apoptosis Detection Kit and Horseradish peroxidase-conjugated (HRP)-labeled Goat Anti-Mouse, Donkey Anti-Goat, and Goat Anti-Rabbit IgG secondary antibodies were purchased from Beyotime Biotechnology (Shanghai, China),

### Cell culture

H9C2 cardiomyocytes were cultured in high-glucose DMEM media supplemented with 10% FBS and 1% streptomycin-penicillin solution (100 ×) and were grown in an incubator at 37 °C with 5% CO_2_.

### Cell transfection

H9C2 cardiomyocytes were seeded in 6-well plates at a density of 5×10^5^ cells/well for 24 h and then transfected with a miR-378a-3p mimic (sequence: 5′-ACUGGACUUGGAGUCAGAAGG-3′), inhibitor (sequence: 5′-CCUUCUGACUCCAAGUCCAGU-3′) or negative control (NC) for 6 h using Lipofectamine 2000 according to the manufacturer’s instructions. Otherwise, H9C2 cardiomyocytes were transfected with three siRNAs (siRNA-1, position 133-151, 5′-GCACTTCTCTGAATTACAA-3′; siRNA-2, position 170-198, 5′-GCAGACCATGGATAACTTA-3′; siRNA-3, position 418-436, 5′-GGAACCTGCTTGTCGAGAA-3′) targeting human TRIM55 (NM_001012218.1) or scramble siRNA (siNC) by using Lipofectamine 2000 following the manufacturer’s instructions. After transfection, the H9C2 cardiomyocytes were further cultured in complete DMEM for 24 h. Full-length human TRIM55 or DUSP1 cloned into the lentiviral expression vector pLVX-Puro (Addgen, Cambridge, MA, USA) was used for TRIM55 or DUSP1 overexpression, while a blank pLVX-Puro vector was used as a negative control (vector). After transfection, the recombined lentiviral vectors were collected and then transduced into H9C2 cardiomyocytes.

Furthermore, full-length DUSP1 and mutant cDNAs were cloned and inserted into the pCMV-Tag 2B vector and the generated plasmids were designated as DUSP1 (WT), DUSP1 (K57R), DUSP1 (K122R), and DUSP1 (K192R). The mutations were introduced into DUSP1 using the QuikChange II Site-directed Mutagenesis kit (Agilent Technologies, Santa Clara, CA, USA). The Myc-tagged TRIM55 sequence was purchased from GENEWIZ, lnc. (Suzhou, China) and cloned into the p-DONR221 vector to express myc-TRIM55. For His-ubiquitin (Ub), human ubiquitin was cloned into the pcDNA-DEST40 vector with a His tag. All constructs and mutants were confirmed by sequencing. HEK293T cells were co-transfected with DUSP1 (WT) or mutant DUSP1 constructs along with the myc-TRIM27 and His-Ub constructs using the Lipofectamine 2000 reagent according to the manufacturer's instructions.

### Simulated I/R protocol

H9C2 cardiomyocytes were cultured in DMEM media at 37°C in an incubator with 5% CO_2_ and 1% O_2_ for 3 h and then transferred into complete DMEM media followed by further incubation for 6, 12, and 24 h.

### Flow cytometry

H9C2 cardiomyocytes (3×10^5^ cells/well) were seeded in 6-well plates. Twenty-four hours after transfection, the cells exposed to ischemia for 3 h and then reperfusion for 24 h were collected by centrifugation at 1200 × g for 5 min, resuspended in 195 μL Annexin V-FITC binding buffer, and then incubated in the dark with 5 μL Annexin V-FITC for 15 min and 5 μL Propidium Iodide for 5 min at 4 °C. Cell apoptosis analysis was performed via flow cytometry (FACSCalibur, BD Biosciences).

### Real-time PCR

Total RNA was extracted from H9C2 cardiomyocytes or rat myocardium following I/R injury using the TRIzol reagent according to the manufacturer’s protocol. Reverse transcription analysis was performed using the M-MLV Reverse Transcriptase. All gene transcripts were measured by real-time PCR using the Applied Biosystems Prism 7300 sequence detection system with the Maxima SYBR Green/ROX qPCR Master Mix according to the manufacturer’s protocol. The primers used were as follows: TRIM55-F: 5′-TTCCAGAGGCAGAAGTCAG-3′; TRIM55-R: 5′-GATGGCTTGGGTCATTTCG-3′. Bax-F: 5′-GGACGCATCCACCAAGAAG-3′; Bax-R: 5′-CTGCCACACGGAAGAAGAC-3′. Bcl-2-F: 5′-GATAACCGGGAGATCGTG-3′; Bcl-2-R: 5′-GGCTGGAAGGAGAAGATG-3′. GAPDH-F: 5′-GGAGTCTACTGGCGTCTTCAC-3′; GAPDH-R: 5′-ATGAGCCCTTCCACGATGC-3′. miR-378a-3p-F: 5′-ACACTCCAGCTGGGACTGGACTTGGAGTC-3′; miR-378a-3p-R: 5′-TGGTGTCGTGGAGTCG-3′. 5S-F: 5′-AGGTGGTCTCCCATCCAAGT-3′; 5S-R: 5′-CTACGGCCATACCACCCTGAAC-3′. GAPDH and 5S RNA were used as mRNA and miRNA internal control, respectively. The relative quantification was performed by using the 2^-**ΔΔ**Ct^ cycle threshold method.

### Western blotting

Total protein was extracted from H9C2 cardiomyocytes or rat myocardium following I/R injury using the RIPA buffer according to the manufacturer’s protocol. Protein concentrations were measured with the BCA Protein Assay kit and then subjected to 10% Sodium dodecyl-sulfate polyacrylamide gel electrophoresis (SDS-PAGE) and transferred to nitrocellulose membranes (Millipore, Burlington, MA, USA). The blots were blocked in 5% non-fat milk overnight at 4 °C and then incubated with anti-TRIM62, anti-DUSP1, anti-p-JNK1/2, anti-JNK1/2, anti-cleaved PARP, anti-cleaved caspase-3, anti-Bax, anti-Bcl-2 or anti-GAPDH antibodies overnight at 4 °C, followed by incubation with appropriate HRP-linked secondary antibodies for 1 h at 37 °C. The membrane was visualized using the ECL kit. The density of each band was quantified using the ImageQuant program and all protein expression levels were evaluated relative to GAPDH expression.

### Dual-luciferase reporter assay

H9C2 cardiomyocytes (5×10^5^ cells/well) were seeded in 6-well plates, cultured at 37°C in a humidified 5% CO_2_ incubator for 24 h, and then co-transfected with 5 μL miR-3568 mimic, inhibitor or negative control (NC) and pGL3-TRIM55-3′ UTR luciferase reporter vector at 37 °C for 6 h using Lipofectamine 2000 according to the manufacturer's instructions. The dual-luciferase reporter assay was performed as previously described [34].

### Co-immunoprecipitation

Whole-cell lysates obtained by centrifugation were incubated with 2 μg anti-TRIM55, anti-DUSP1 or normal IgG antibodies and protein G-Agarose beads (Roche Diagnostics Ltd, Shanghai, China) at 4 °C overnight. The immunocomplexes were then separated by SDS-PAGE and blotted with the indicated antibodies.

### Ubiquitination assay

H9C2 cardiomyocytes were transfected with TRIM55-siRNA or siNC and the lysates were subjected to immunoprecipitation with IgG or anti-DUSP1 antibodies overnight at 4 °C. Bound proteins were released from the protein G-Agarose beads by boiling in SDS-PAGE sample buffer followed by immunoblotting with an anti-Ub antibody.

### His-Ub pull-down assay

H9C2 cardiomyocytes were co-transfected with Flag-tagged DUSP1 (WT) or mutant DUSP1 constructs along with the myc-TRIM55 and His-Ub constructs. Forty-eight hours after transfection, the cells lysates were incubated with Ni2+-NTA agarose beads (Qiagen). The washed complexes were eluted by boiling in SDS sample buffer and then separated by SDS-PAGE. The protein interactions were then analyzed by western blotting.

### Rat myocardial I/R model

The myocardial I/R injury model in Sprague-Dawley rats (10-12 weeks old, weighing 350 ± 25 g) was performed via left coronary artery (LCA) ligation for 20 min and then reperfusion for 2 h. A dose of 50 mg/kg of miR-378a-3p mimic or NC was injected into the left ventricular anterior wall 24 h prior to I/R. Rats with a sham operation without LCA ligation were used as a control. When the experiment ended, rats were anaesthetized and the myocardial tissue was harvested, stained with hematoxylin and eosin (H&E), and incubated with terminal-deoxynucleotidyl transferase-mediated nick end labeling (TUNEL) as previously described [35]. The present study was performed in strict accordance with the guidelines on ethical care for experimental animals and approved by the Animal Research Committee of the Huashan Hospital.

### Statistical analysis

Each experiment was performed in triplicate and the data were presented as the mean ± standard error of the mean (SEM). Statistical analyses were done using the GraphPad Prism software Version 6.0 (San Diego, CA, USA) with the Analysis of Variance (ANOVA) test. The criterion for statistical significance was set at *P* < 0.05.
